# Deciphering the Lipid-Random Copolymer Interactions and Encoding Their Properties to Design a Hybrid System

**DOI:** 10.1021/acs.langmuir.4c00278

**Published:** 2024-05-27

**Authors:** Efstathia Triantafyllopoulou, Aleksander Forys, Diego Romano Perinelli, Anastasia Balafouti, Maria Karayianni, Barbara Trzebicka, Giulia Bonacucina, Georgia Valsami, Natassa Pippa, Stergios Pispas

**Affiliations:** † Section of Pharmaceutical Technology, Department of Pharmacy, School of Health Sciences, National and Kapodistrian University of Athens, Panepistimioupolis Zografou, Athens 15771, Greece; ‡ Centre of Polymer and Carbon Materials, Polish Academy of Sciences, Zabrze 41-819, Poland; § School of Pharmacy, University of Camerino, Via Gentile III da Varano, Camerino 62032, Italy; ∥ Theoretical and Physical Chemistry Institute, National Hellenic Research Foundation, 48 Vassileos Constantinou Avenue, Athens 11635, Greece; ⊥ Department of Pharmaceutical Technology, Faculty of Pharmacy, National and Kapodistrian University of Athens, Panepistimioupolis Zografou 15771, Athens 157 72, Greece

## Abstract

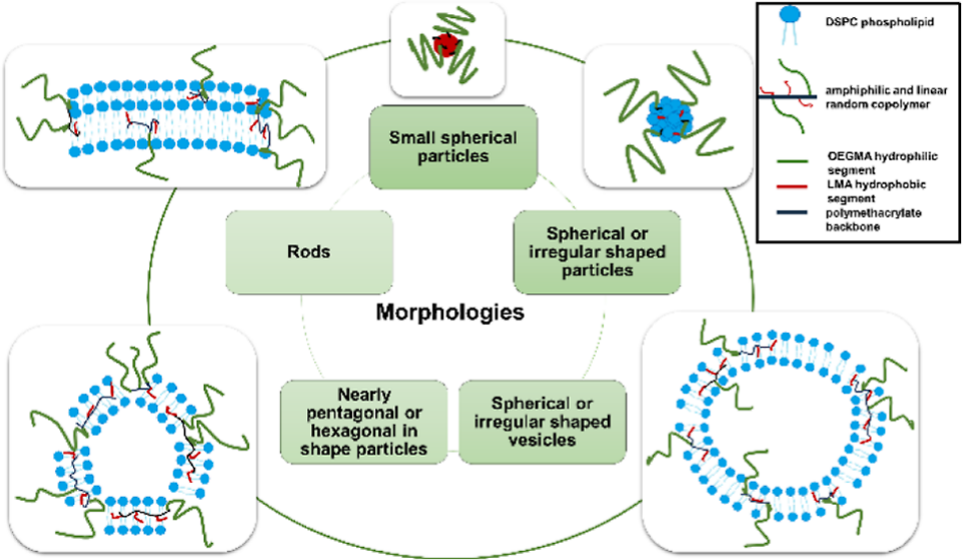

Lipid/copolymer colloidal systems are deemed hybrid materials with unique properties and functionalities. Their hybrid nature leads to complex interfacial phenomena, which have not been fully encoded yet, navigating their properties. Moving toward in-depth knowledge of such systems, a comprehensive investigation of them is imperative. In the present study, hybrid lipid/copolymer structures were fabricated and examined by a gamut of techniques, including dynamic light scattering, fluorescence spectroscopy, cryogenic transmission electron microscopy, microcalorimetry, and high-resolution ultrasound spectroscopy. The biomaterials that were mixed for this purpose at different ratios were 1,2-dioctadecanoyl-*sn*-glycero-3-phosphocholine and four different linear, statistical (random) amphiphilic copolymers, consisting of oligo(ethylene glycol) methyl ether methacrylate as the hydrophilic comonomer and lauryl methacrylate as the hydrophobic one. The colloidal dispersions were studied for lipid/copolymer interactions regarding their physicochemical, morphological, and biophysical behavior. Their membrane properties and interactions with serum proteins were also studied. The aforementioned techniques confirmed the hybrid nature of the systems and the location of the copolymer in the structure. More importantly, the random architecture of the copolymers, the hydrophobic-to-hydrophilic balance of the nanoplatforms, and the lipid-to-polymer ratio are highlighted as the main design-influencing factors. Elucidating the lipid/copolymer interactions would contribute to the translation of hybrid nanoparticle performance and, thus, their rational design for multiple applications, including drug delivery.

## 1 Introduction

Nanoparticles as drug delivery systems belong to a widely growing sector in pharmaceutical technology.^1,2^ Hybrid nanoparticles composed of two or more distinct components, and especially hybrid lipid/polymer nanoparticles, are a hopeful candidate for drug delivery purposes, among others.^3−12^ Even though the utilization of marketed available polymers and block copolymers is very common for this purpose, there are not a lot of such examples utilizing statistical (random) copolymers.^9,10,13^ Statistical (random) copolymers are comprised of two different comonomers which are randomly dispersed, whereas amphiphilic copolymers can self-assemble into intra- or multichain aggregates and can be tailor-made according to intended needs.^14−16^ Thus, the combination of amphiphilic random copolymers with lipids could lead to nanocarriers of great interest with novel intrinsic features.

However, knowledge of such hybrid systems is still limited. Since there are still considerations in general for nanoparticles regarding toxicity, reproducibility, and scale-up procedures, the exploration of the parameters affecting their performance is still of great importance for the in-depth understanding of these systems and for their optimization as well.^1,2,10,17,18^ Besides, the regulation framework dictates well-defined nanoformulations, well-characterized by multiple/complementary techniques, so as to prove their structure, properties, safety, and effectiveness.^2,19^ Techniques that are suggested for this purpose include dynamic light scattering (DLS) for physicochemical evaluation, differential scanning calorimetry (DSC or microDSC) for thermodynamic investigation, and cryogenic transmission electron microscopy (cryo-TEM) for examination of their morphology. Fluorescence spectroscopy (FS) is another useful technique for exploring lipid and lipid/copolymer nanoparticle coassembly properties. In fact, the membrane mechanics of such systems could be investigated by utilizing the Laurdan probe.^20−26^ Laurdan is a hydrophobic probe which can be embedded into the hydrophobic parts of the lipid/polymer nanoparticles and elicit information about their internal microfluidity.^27^

The necessity of examining the features of hybrid nanoparticles, and especially the interactions between the different biomaterials used each time by a variety of methodologies, comes from the fact that these interactions significantly influence the main aspects of the hybrid systems’ performance. First, the lipid/polymer interactions have a great impact on the structure of hybrid systems. Their coassembly could affect their internal and external morphology and even result in compartmentalized structures. Moreover, they could influence the physicochemical properties of the nanoparticles as a whole, including size, size distribution, and zeta potential (nanoparticle surface charge). These characteristics are crucial for the administration route of choice, hybrid nanoparticle therapeutic efficacy, and their intrinsic toxicity, among others. The physical stability of such colloidal dispersions is another important factor resulting from hydrophobic, electrostatic, and solvation forces. Besides, the interactions between the lipids and polymers lead to specific surface properties that are mainly responsible for nanoparticle interactions with serum proteins and the overall fate of the nanostructures in vivo. Concerning their role as drug delivery vehicles, the encapsulation efficacy of the active pharmaceutical ingredient as well as the drug release profile could be influenced significantly.

In this work, hybrid lipid/copolymer colloidal dispersions comprising 2-dioctadecanoyl-*sn*-glycero-3-phosphocholine (DSPC) as the lipid part and a statistical (random) copolymer as the polymeric part were prepared by the thin film hydration method. Four different amphiphilic and linear copolymers were used, consisting of oligo(ethylene glycol) methyl ether methacrylate (OEGMA) as the hydrophilic comonomer and lauryl methacrylate (LMA) as the hydrophobic one. The copolymers differ in the comonomer ratio and length of the OEGMA chains. Our previous studies on lipid/copolymer systems^28^ and the information that was gained guided us to select the most promising hybrid platforms for further investigation. The selection of the systems is based on thermodynamic, physicochemical, and toxicological criteria in order to encode the lipid-copolymer interactions and the crucial design parameters affecting their performance. Briefly, the increase of the P(OEGMA_950_-*co*-LMA) content (31% LMA component) into the DSPC-containing hybrid nanostructures was accompanied by increased biocompatibility and unique physicochemical characteristics. Besides, the incorporation of different P(OEGMA-*co*-LMA) copolymers with varying hydrophilic to hydrophobic ratio into DSPC hybrid systems in a constant 9:1 lipid-to-polymer weight ratio favored different nanotoxicity profiles and highlighted the importance of coassembly to the biocompatibility of the nanostructures. In general, the nanoplatforms of choice had an appropriate size for drug delivery applications (less than 200 nm) and were nontoxic. Most of them were stable for 28 days as well (unpublished data). The main goal of the present study is to decipher the design factors that mainly influence the membrane properties, the thermodynamic features, the adsorption of serum proteins, and the morphology of lipid/random copolymer structures driven by the complex interactions between lipids and copolymers. For this purpose, the systems were thoroughly examined by a gamut of techniques, including DLS, FS, mDSC, high-resolution ultrasound spectroscopy (HR-US), and cryo-TEM. To the best of the authors’ knowledge, this is the first time that DSPC:P(OEGMA-*co*-LMA) hybrid particles are examined for their colloidal behavior from the perspective of the membrane’s fluidity, the biophysics, the morphology, and their interactions with serum proteins utilizing the aforementioned techniques.

## 2 Experimental Section

### 2.1 Materials

**Figure 1:**
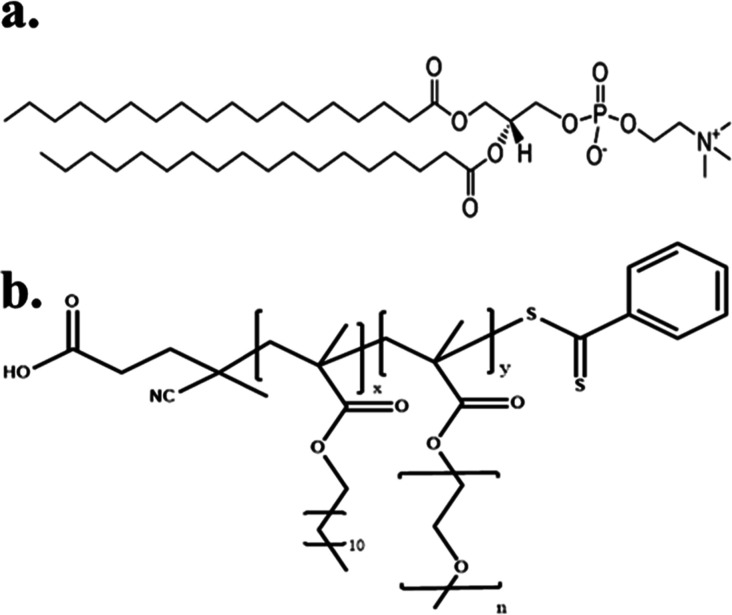
Chemical structure of the utilized biomaterials: (a) DSPC and (b) linear random copolymer P(OEGMA-*co*-LMA).

DSPC phospholipid was purchased from Lipoid GmbH, while all the reagents were from Sigma-Aldrich Chemical Co., St. Louis, MO, USA, and were of analytical grade. The random copolymers used were synthesized in-house by the RAFT polymerization method, and their chemical composition as well as their % comonomer ratios are summarized in Table 1. The aforementioned biomaterials were mixed in selected combinations, and their chemical structures are illustrated in Figure 1. The fluorescent probe 6-dodecanoyl-*N*,*N*-dimethyl-2-naphthylamine (Laurdan) was also purchased from Sigma-Aldrich Chemical Co., St. Louis, MO, USA.

**Table 1:** Chemical Characteristics of the Statistical (Random) Copolymers Used in This Study

copolymer	abbreviation	*M*_w_^a^ (×10^4^) (g/mol)	*M*_w_/*M*_n_^b^	% P(OEGMA)^c^
P(OEGMA_950_-*co*-LMA)^d^	copolymer 1	1.25	1.11	69
P(OEGMA_950_-*co*-LMA)	copolymer 2	1.32	1.12	51
P(OEGMA_500_-*co*-LMA)	copolymer 3	1.00	1.17	64
P(OEGMA_500_-*co*-LMA)	copolymer 4	1.22	1.16	53

a By ^1^H NMR in CDCl_3_.

b By GPC in THF at 25 °C.

c OEGMA: oligo(ethylene glycol) methyl ether methacrylate (hydrophilic comonomer).

d LMA: lauryl methacrylate (hydrophobic comonomer).

### 2.2 Methods

#### 2.2.1 Preparation of Lipid/Copolymer Hybrid Systems

The thin film hydration protocol was used for lipid/copolymer hybrid system formation, as described in detail elsewhere.^29^ The colloidal concentration in all cases was equal to *C* = 5 mg/mL. The colloidal concentration refers to the total concentration of the biomaterials in an aqueous medium. As a continuation of our previous publications, the selected systems that were prepared are DSPC/P(OEGMA-*co*-LMA)-1 at three different lipid to polymer weight ratios (9:1, 7:3, and 5:5), and DSPC/P(OEGMA-*co*-LMA)-2, DSPC/P(OEGMA-*co*-LMA)-3, and DSPC/P(OEGMA-*co*-LMA)-4 at a lipid to polymer weight ratio of 9:1.

#### 2.2.2 Dynamic Light Scattering

The physicochemical characteristics of the systems, the scattered light intensity (*I*), the hydrodynamic radius (*R*_h_), and the size polydispersity index (PDI), were evaluated by the DLS technique the day of their preparation and during a period of 28 days. For this purpose, 50 μL of each dispersion were diluted with 2 mL of HPLC-grade water. The measurements were conducted at a fixed scattering angle of 90° and at ambient temperature, while they were analyzed using the CONTIN algorithm. Each experiment involved 5 measurements and was performed on three independent samples. The stability study simulating in vivo conditions was carried out at body temperature, and the dispersion medium was comprised of fetal bovine serum and phosphate buffered saline (FBS and PBS, respectively) at a weight ratio FBS/PBS of 9:1. The equilibration period was 5 min. The information on the equipment that was utilized for nanoparticles’ characterization can be found in the literature.^29^

#### 2.2.3 Fluorescence Spectroscopy

The fluorescence spectra collected give information about the membrane properties, specifically on the microfluidity of the hybrid systems utilizing Laurdan as the hydrophobic probe. The fluorescence intensity measurements took place at room temperature with a double-grating excitation and a single grating emission spectrofluorometer (Fluorolog-3, model FL3-21, Jobin Yvon-Spex, Horiba Ltd., Kyoto, Japan). The hybrid nanoparticle dispersions were inserted into a quartz cell, and detection was done at a 90° angle relative to the incoming beam direction. The thermoresponsive systems were recorded also at 37 °C. For each experiment, three independent samples were measured for statistical reasons. The emission spectra were recorded from λ_em_ = 380–600 nm using an excitation wavelength of λ_ex_ = 340 nm. The general polarization value (GP) was calculated using the following equation
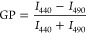
1where *I*_440_ and *I*_490_ are the intensities at the blue and red edges of the emission spectrum, respectively. The Laurdan probe was diluted in ethanol, and the stock solution had a final concentration of 0.5 mM. The protocol that was followed by the mixing of 1 mL of the sample with 5 μL of the probe from the stock solution and a 24 h rest period (4 °C) before the insertion into the sample cell. For samples that were tested at 37 °C a 5 min incubation time preceded the measurement.

#### 2.2.4 Cryogenic Transmission Electron Microscopy

cryo-TEM images were obtained using a Tecnai F20 X TWIN microscope (FEI Company, Hillsboro, Oregon, USA) equipped with a field emission gun, operating at an acceleration voltage of 200 kV. Images were recorded on the Gatan Rio 16 CMOS 4k camera (Gatan Inc., Pleasanton, California, USA) and processed with Gatan Microscopy Suite (GMS) software (Gatan Inc., Pleasanton, California, USA). Specimen preparation was done by vitrification of the aqueous solutions on grids with a holey carbon film (Quantifoil R 2/2; Quantifoil Micro Tools GmbH, Großlöbichau, Germany). Prior to use, the grids were activated for 15 s in oxygen plasma using a Femto plasma cleaner (Diener Electronic, Ebhausen, Germany). Cryo-samples were prepared by applying a droplet (3 μL) of the suspension to the grid, blotting with filter paper, and immediately freezing in liquid ethane using a fully automated blotting device, Vitrobot Mark IV (Thermo Fisher Scientific, Waltham, Massachusetts, USA). After preparation, the vitrified specimens were kept under liquid nitrogen until they were inserted into a cryo-TEM-holder Gatan 626 (Gatan Inc., Pleasanton, USA) and analyzed by TEM at −178 °C.

#### 2.2.5 Microcalorimetry

Calorimetric analyses were conducted in the colloidal state of the hybrid systems via a microDSC III instrument (Setaram, France). The melting temperature (*T*_m_, °C) and corresponding enthalpy (Δ*H*, J/g of solution) were calculated by utilizing the software of the instrument (Setsoft2000, Setaram) according to the tangent method. All measurements were performed in the temperature range of 5 to 80 °C in triplicate and the exact protocol can be found in other studies.^30,31^

#### 2.2.6 High-Resolution Ultrasound Spectroscopy

HR-US is used to investigate the performance of highly structured systems without further dilution. Ultrasound parameters were collected as a function of the temperature using an HR-US 102 high-resolution spectrometer (Ultrasonic Scientific, Ireland). Ultrasonic cells were filled with 2 mL of sample, and the reference was HPLC-grade water. The thermal program of choice was the same for the mDSC analyses.^32^

## 3 Results and Discussion

### 3.1 Physicochemical Characterization of Lipid/Copolymer Colloidal Dispersions

**Figure 2:**
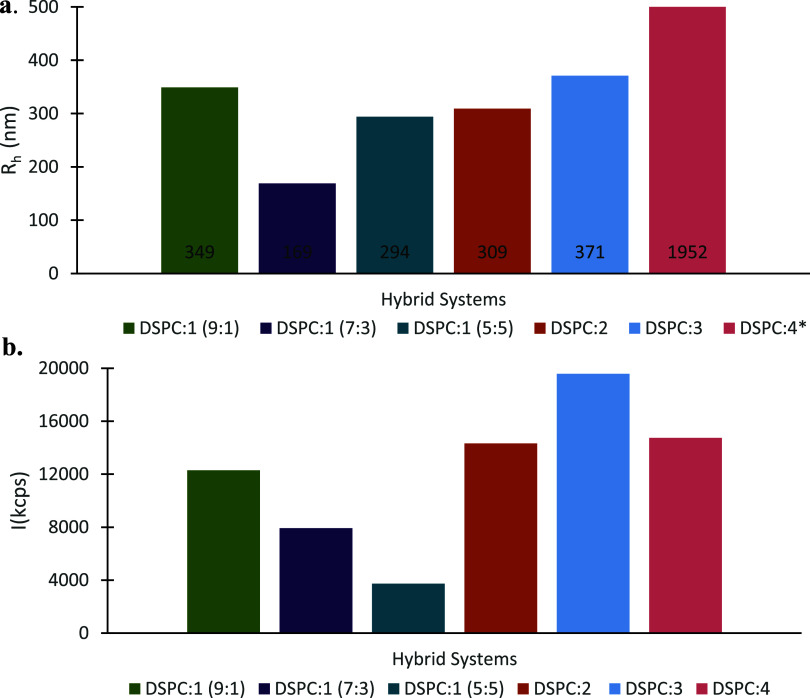
Physicochemical characteristics: (a) hydrodynamic radius (*R*_h_, nm) and (b) intensity (kilocounts per second, or kcps) of different hybrid systems in water for injection dispersion medium and at ambient temperature. The standard deviation (SD) in both diagrams is less than 10%. *DSPC:4 hybrid system has a very high *R*_h_ compared to the rest of the systems that exceeds the scale of the graph.

**Figure 3:**
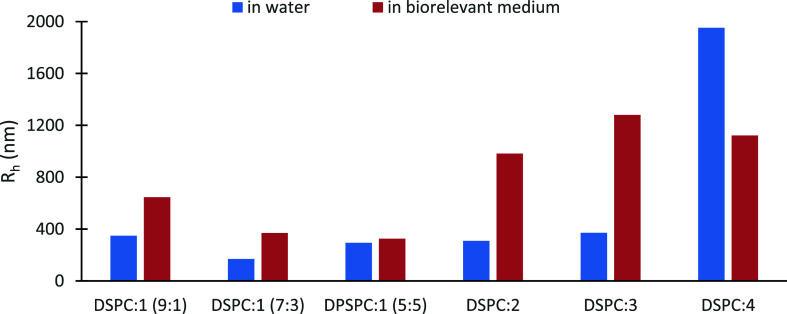
Hydrodynamic radius (*R*_h_, nm) of hybrid systems in different dispersion media at 37 °C. The standard deviation (SD) is less than 10%.

The prepared lipid-copolymer systems were examined via dynamic light scattering and fluorescence spectroscopy to elucidate their properties. Physicochemical evaluation was conducted the day of their preparation in aqueous medium (in particular water for injection) at ambient temperature and in biorelevant medium—namely, FBS/PBS 9:1 weight ratio—at body temperature. Stability assessment took place in water as an injection medium for a period of 28 days as well (data not shown). The results are summarized in Tables S1 and S2 and illustrated in Figures 2, 3, and S1.

**Figure 4:**
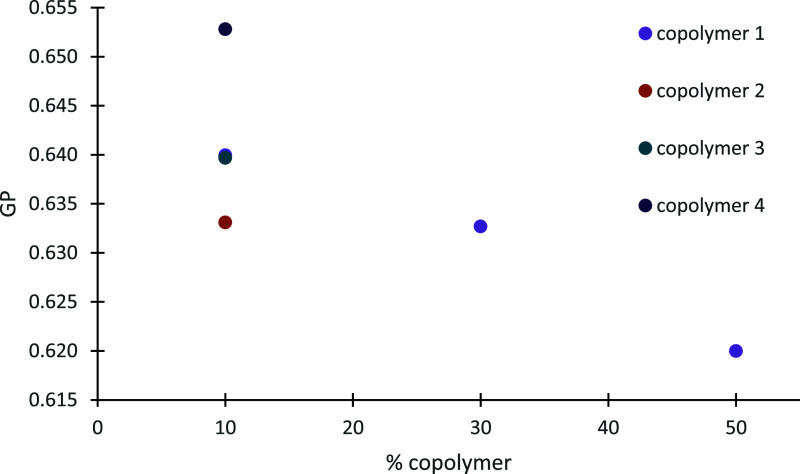
GP values derived from Laurdan fluorescence spectra vs % copolymer of DSPC hybrid nanostructures. The standard deviation (SD) is less than 10%.

For FS measurements, the Laurdan probe was utilized to investigate the microfluidity of the particles, and the results appear in Table S1 and Figure 4. In particular, Laurdan is sensitive to polarity fluctuations, emitting in a more red-shifted spectrum range. The differences in membrane fluidity can be translated to a semiquantitative parameter—the general polarization (GP). A higher GP value reflects a more ordered and less fluid bilayer.^33^

The presence of P(OEGMA-*co*-LMA) copolymers in the colloidal dispersions did not bring about any specific patterns as far as the physicochemical characteristics of the hybrid systems are concerned, despite the differences in the length of the OEGMA side chain (OEGMA_950_ or OEGMA_500_) and/or the %LMA content in the copolymers (Table 1). The exceptional behavior of such a mixed system due to random copolymer architecture has been reported in our recent studies^28^ and is confirmed in the current one as well. It is worth mentioning, though, that there is a decreasing trend for scattered intensity by increasing the % weight P(OEGMA-*co*-LMA)-1 in the hybrid nanoparticles (Table S1 and Figure 2b), which overall is proportional to the mass of the colloidal systems.^34,35^ There is not an equivalent pattern for the size, though. Moreover, the systems tend to be rather polydisperse, showing PDI values between 0.33 and 0.49 from DLS, accompanied by an *R*_h_ from 170 to 370 nm. However, the incorporation of copolymer-4 into the hybrid structure is characterized by an enormous size on the scale of micrometers in combination with a small scattered light intensity (Table S1 and Figure 2). In our opinion, this is a sign of loose aggregate formation in accordance with cryo-TEM results. Additionally, the moderate to high PDI values could be accredited to the variety of hybrid morphologies that were observed in cryo-TEM images (see also Exploring the Morphology of Lipid/Copolymer Structures Section).

All the hybrid systems correspond to similar GP values with slight differences; in particular, GP ranges between 0.620 and 0.653 at ambient temperature (Table S1), although there are some trends that should be mentioned. First, DSPC hybrid systems of a similar but low % LMA content [incorporating P(OEGMA-*co*-LMA)-1 or P(OEGMA-*co*-LMA)-3 in a 9:1 lipid to polymer weight ratio] have the same GP value (0.640) regardless of the OEGMA chain length, indicating an ordered lipid bilayer due to LMA interference into it, probably in a small amount. This finding is an indication that the OEGMA chains are located in the outer region, interacting with the solvent, and only the LMA chains penetrate into the bilayer. Noticeably, it seems that there is a hydrophobic threshold, and more addition of LMA chains into the bilayer might lead to chain mismatch and disorder, as the GP index indicates in the case of copolymer-2 (0.633). The disconformation and reduction of the rigidity of the bilayer by increasing LMA chain proportion is also in good agreement with the GP value of the DSPC/P(OEGMA-*co*-LMA)-1 system; GP decreases according to a lipid-to-polymer dependent manner, as can be seen in Figure 4. The GP value of DSPC:P(OEGMA-*co*-LMA)-4 is the highest one (0.653). It is our hypothesis that this could be the result of the aggregation phenomena that were observed by DLS and cryo-TEM techniques.

It is well known that the interactions of nanomaterials with serum proteins lead to the formation of the protein corona, which is a new in vivo morphology with unique characteristics.^36−38^ The protein corona influences the fate of the nanovectors and, in most cases, accredits them to the opsonization effect, and thus to their rapid removal from the body or their coagulation.^39−42^ In this manner, we investigated the response of the selected systems to biorelevant solution conditions. As can be seen in Table S2 and Figure 3, all the hybrid nanoplatforms adsorb serum proteins, and this is illustrated by a size increase and a concurrent heterogeneous size distribution. The intensity values varied, but, in most cases, an increase is observed as well (indicating an increase in the mass of the dispersed species). The only exception regards the DSPC:P(OEGMA-*co*-LMA)-1 5:5 mixed system, which maintains its size despite the small increase in the mass of the colloidal system. This system may be indicated as stealthy due to no protein corona formation, at least in vitro. Stealth systems have the ability to remain in the bloodstream for a longer period of time, avoiding the reticuloendothelial system. Generally, the OEGMA chains on the exterior of the hybrid nanoparticles could ameliorate the in vivo behavior of the hybrid systems and impart nonfouling properties to the nanoparticles. However, the length and density of the OEGMA chains play a crucial role in their configuration and thus their functionality.^43−46^ The protein corona formation and its results in vivo are a multifactorial concept that is affected by many parameters, including size and chemical composition.^42,47−50^ We are convinced that the random architecture of the copolymers utilized in this study, as well as the hydrophobic-to-hydrophilic balance, have a great impact on protein adsorption, and to the best of our knowledge, this is the first time that this has been reported.

Investigating the stability of the colloidal systems, we could conclude that most of the systems did not manage to maintain their physicochemical properties for a period of 28 days. However, the majority of studied hybrid platforms, such as DSPC:P(OEGMA-*co*-LMA)-2 and DSPC:P(OEGMA-*co*-LMA)-1 (9:1, 7:3, and 5:5) systems, were stable for at least 1 or 2 weeks in storage conditions, and this is probably attributed to the long OEGMA_950_ chains offering steric stabilization. Even though the results of this study advocate extensive protein binding and the instability of the hybrid structures for more than 14 days, we believe that this outcome is directly related to the scale-up of the preparation method, and it would be further explained in the Conclusion Section.

### 3.2 Exploring the Morphology of Lipid/Copolymer Structures

**Figure 5:**
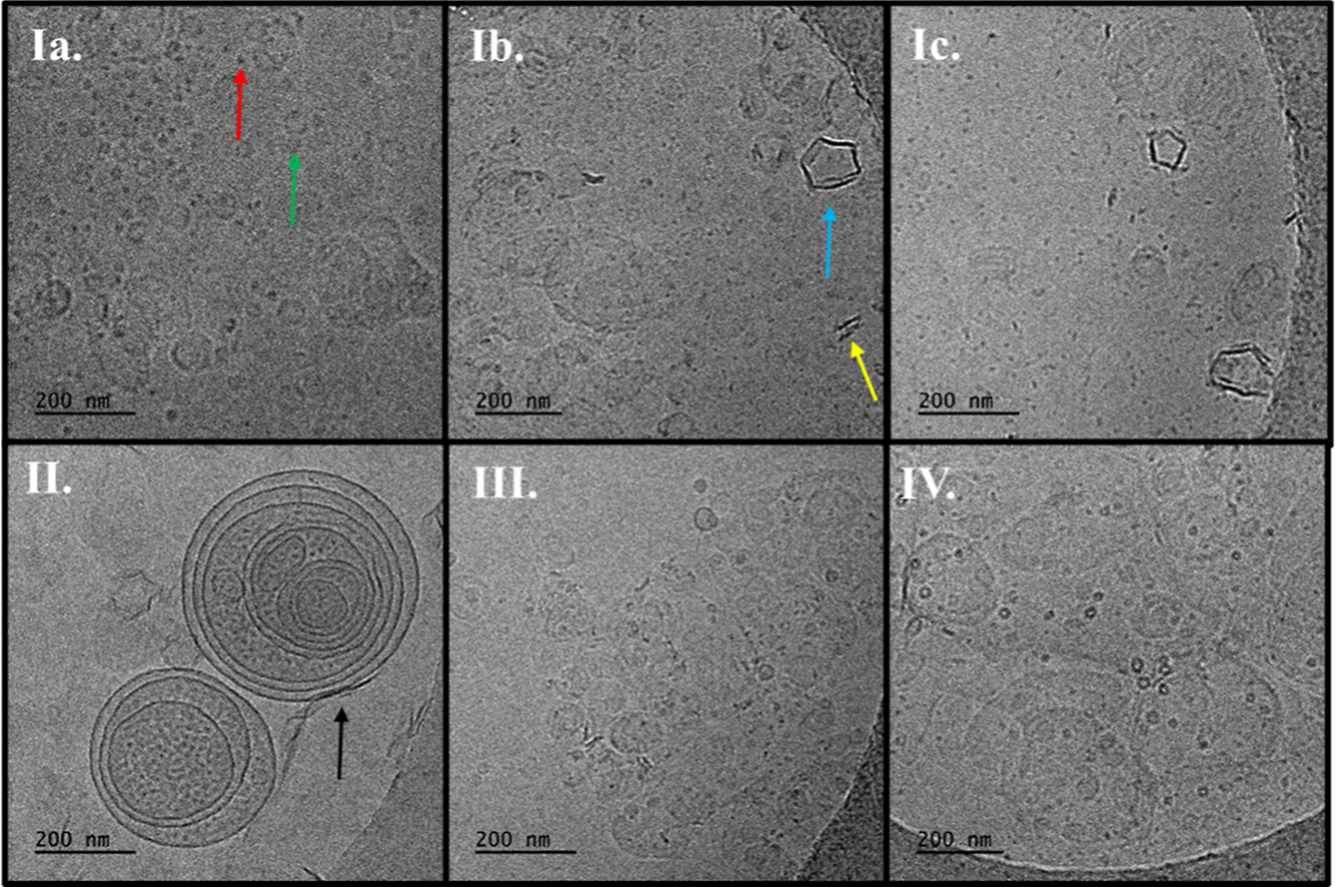
Cryo-TEM images of DSPC:P(OEGMA-*co*-LMA) hybrid systems of the different comonomer ratio (varying % PLMA) and/or different oligoethylene glycol side chain length (OEGMA_950_ or OEGMA_500_): (I) DSPC:copolymer-1 in different lipid to polymer weight ratios: a. 9:1, b. 7:3, and c. 5:5 ratio, (II) DSPC:copolymer-2, (III) DSPC:copolymer-3, and (IV) DSPC:copolymer-4. The colored arrows point out a different morphology; namely small spherical particles (red arrow), spherical, or irregular shape particles with distinct membranes (green arrow), rods (yellow arrow), nearly pentagonal-shaped particles (blue arrow), and spherical or irregular-shaped vesicles (black arrow).

The hybrid platforms were also examined via cryo-TEM, and their morphology is illustrated in Figure 5. The prepared lipid/copolymer systems exhibited a gamut of different structures, which are summarized in Table 2 along with their characteristic dimensions.

**Table 2:** Dimensional Properties of Objects Formed by Different Lipid/Copolymer Systems Measured by Cryo-TEM

sample	L/P weight ratio	type of objects	wall thickness or core diameter for rods^a^ (nm)	diameter or length for rods^a^ (nm)
DSPC:copolymer 1	9:1	small spherical particles		8–15
		spherical or irregular-shaped particles	8–10	20–300^b^
		rods	8–10	30–60
DSPC:copolymer 1	7:3	small spherical particles		8–15
		spherical or irregular-shaped particles	8–10	20–400^b^
		rods	8–10	30–70
		nearly pentagonal-shaped particles	8–10	
DSPC:copolymer 1	5:5	small spherical particles		8–15
		spherical or irregular-shaped particles	8–10	20–300^b^
		rods	8–10	20–40
		nearly pentagonal-shaped particles	8–10	
DSPC:copolymer 2	9:1	small spherical particles		8–15
		spherical or irregular-shaped particles	8–10	30–800^b^
		spherical or irregular-shaped vesicles	6	40–500
		rods	8–10	60–150
		nearly pentagonal-shaped particles	8–10	
DSPC:copolymer 3	9:1	small spherical particles		8–15
		spherical or irregular-shaped particles	8–10	20–300^b^
		spherical or irregular-shaped vesicles	6	30–100
		rods	8–10	20–60
		nearly pentagonal-shaped particles	8–10	
DSPC:copolymer 4	9:1	small spherical particles		8–15
		spherical or irregular-shaped particles	8–10	20–500^b^
		spherical or irregular-shaped vesicles	6	20–80
		rods	8–10	20–40

a Average size observed for 50 objects.

b It is difficult to determine the upper limit of sizes due to the irregular shape of particles.

The morphological diversity of colloidal dispersions has been mentioned before.^30,51,52^ One of the main driving forces for this phenomenon could be the membrane’s line tension, which favors the formation of different-shape structures due to the thickness mismatch between copolymers and phospholipids.^53^ In living cells, the liquid-ordered and disordered domains that are created lead to vesicle formation for exocytosis purposes.^54^ Despite the fact that there is variability in shape, the membrane thickness is constant for most of the morphologies and equals 8–10 nm. This finding suggests that the copolymers were effectively incorporated into the lipid bilayer, and mixed structures were created.^30,51,53,55^ Muller et al.^56^ confirmed the increase of membrane thickness due to different configuration of the copolymer in a lipid-to-polymer ratio-dependent manner. In this study, the membrane thickness does not seem to be influenced by the % weight of the copolymer in the systems. In our opinion, this is attributed to the dominance of the lipids, even at a 5:5 weight ratio, as well as to the random topology of the copolymers. Except for the systems integrating P(OEGMA-*co*-LMA)-1, there are also spherical or irregular-shaped vesicles of 20–100 nm with a thinner membrane and a better imaging contrast of 6 nm—still a little larger than that of neat liposomes (≤5 nm) evidencing a hybrid bilayer with a different conformation of the copolymer.^56−58^ However, Dao et al. (2017) indicated that pure DPPC vesicles were accompanied by a membrane of 6 nm.^51^ Reasonably, we could not exclude the scenario that the membrane of these structures corresponds to neat lipid bilayers. In general, all different systems were composed of small spherical particles, spherical or irregular-shaped particles, and rods. The size of small spherical particles is about 8 to 15 nm. Taking into consideration that amphiphilic random copolymers form single- or multi-chain aggregates, this morphology could refer to neat polymeric micelles.^14−16^ Concerning the rod-like structures, they have a length ranging from 20 to 70 nm and are mentioned in the literature as disk-shaped particles oriented in an edge-on state.^59,60^ They are very common in lipid/polymer nanoparticles, especially in PEGylated liposomes.^52,61−64^ According to the literature, the creation of this structure is the result of biomaterials, which prefer the spontaneous formation of micelles; this configuration is adopted in the interior of bilayers in a temperature-dependent manner, leading to liposome-like but open morphologies.^59,61^ Taking into consideration the amphiphilic copolymers’ tendency to self-assemble into micelles in an aqueous environment,^15,65^ the appearance of rod-like structures could be the result of the preparation procedure, which took place above the main transition temperature (*T*_m_) of the lipid biomaterials.

The differences between the hybrid systems involve mainly size fluctuations or additional configurations. Namely, the presence of P(OEGMA-*co*-LMA)-1 caused the formation of the three major structures that were discussed before (Table 2), while by increasing the copolymer proportion in the bilayer, shaped particles were created (Figure 5Ib, blue arrow). The angular objects are well established in lipid/polymer systems and could be the outcome of microdomains or rafts due to the inhomogeneous distribution of the random copolymer into the bilayer.^52,61^ The hybrid systems of this composition were the only systems that were not composed of vesicles. Conversely, all of the aforementioned morphologies, including pentagonal particles, were observed by utilizing P(OEGMA-*co*-LMA)-2. Interestingly, the DSPC/P(OEGMA-*co*-LMA)-2 systems form larger morphologies compared to the rest of the systems, including giant multivesicular vesicles, as they are called, accompanied by a reticular patchy morphology in the interior region.^66^ The dot-like structure looks like a dense core of hydrophobic polymeric chains that are collapsed, resembling, in a way, a core–shell hybrid structure.^67^ This may be related to the hydrophobic LMA segments building up aggregates due to van der Waals interactions. Namely, the hydrophobic attractive forces may result in a more compact structure of LMA cores in the hybrid systems.^68^ This morphology might affect the drug loading and release kinetics. The presence of P(OEGMA_500_-*co*-LMA) in different %LMA did not reveal any specific characteristics. It is worth mentioning though that DSPC/P(OEGMA-*co*-LMA)-4 dispersion lacks faceted particles, whereas aggregation phenomena probably took place, as can be seen in Figure 5IV, and this is in accordance with the physicochemical results.

Overall, there are some interesting observations concerning the polygonal-shaped particles. It is our hypothesis that there is a correlation between the hydrophobicity level and angularly-shaped particles. Specifically, the incorporation of copolymer-1 in increasing amounts into the hybrid structures leads to the formation of such particles as well as the incorporation of copolymers 2 and 3 in a constant lipid-to-polymer weight ratio (9:1). However, it seems that there is a hydrophobic threshold, and beyond that, the pentagonal-shaped particles are absent, such as in the case of copolymer 4. This could also be the outcome of the shorter and fewer OEGMA chains resulting in a different conformation in the outer aqueous environment.^15,44^ In general, the interfacial curvature can modulate the shape of the nanoparticles depending on the nature of the biomaterials and the critical packing parameter.^55,69^ Random amphiphilic copolymers may not exhibit an exact geometrical critical packing parameter in relation to the macromolecular hydrophobic-to-hydrophilic ratio because of the random distribution of the hydrophobic segments within the polymer chain.^65,70^ In this way, it is our belief that the random architecture of the present copolymers, in combination with their different hydrophobicities, results in a unique hydrophilic-to-hydrophobic balance for each system that dictates the interactions between the biomaterials as well as the interfacial and entropic phenomena, leading to coassembly in an exceptional manner. Besides, the ability of copolymer architecture to affect the membrane’s line tension and the extent of this influence on hybrid system shape have been mentioned before in block copolymers.^51,57^

Interestingly, DLS results are typically more comparable to those of spherical/irregular-shaped particles, as measured by cryo-TEM. In some cases, the size of the particles is smaller than DLS measurements. This is partially due to the difficulty of cryo-TEM to ascertain the upper limit of size in irregular shapes of nanoassemblies. On the other hand, this could also be the result of the OEGMA hydrophilic polymeric chains that are located toward the outer aqueous environment and cannot be detected by this imaging technique compared to light scattering.^52,59^ We should always keep in mind that cryo-TEM can trap intermediate structures via vitrification, compared to the light scattering method, which is applied under real solution conditions and gives information about the particle size on average from an entire sample.^71^ However, the heterogeneous size distribution is reflected in the morphological and dimensional variability. To the best of our knowledge, this is the first report evaluating the morphological characteristics of hybrid particles of DSPC and P(OEGMA-*co*-LMA) biomaterials, while the literature of combining lipids to statistical copolymers, that is, with random architecture/random sequence of hydrophobic/hydrophilic segments, is limited as well.

### 3.3 Thermal Analysis of Hybrid Lipid/Copolymer Colloidal Dispersions

**Figure 6:**
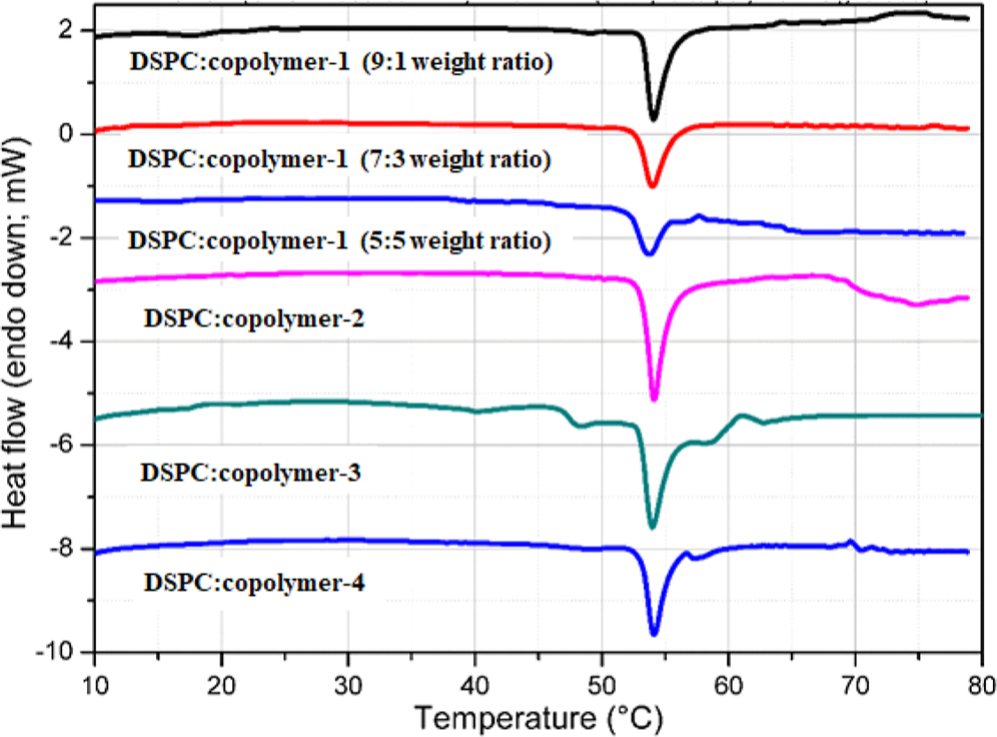
mDSC traces of different DSPC/P(OEGMA-*co*-LMA) hybrid systems.

**Figure 7:**
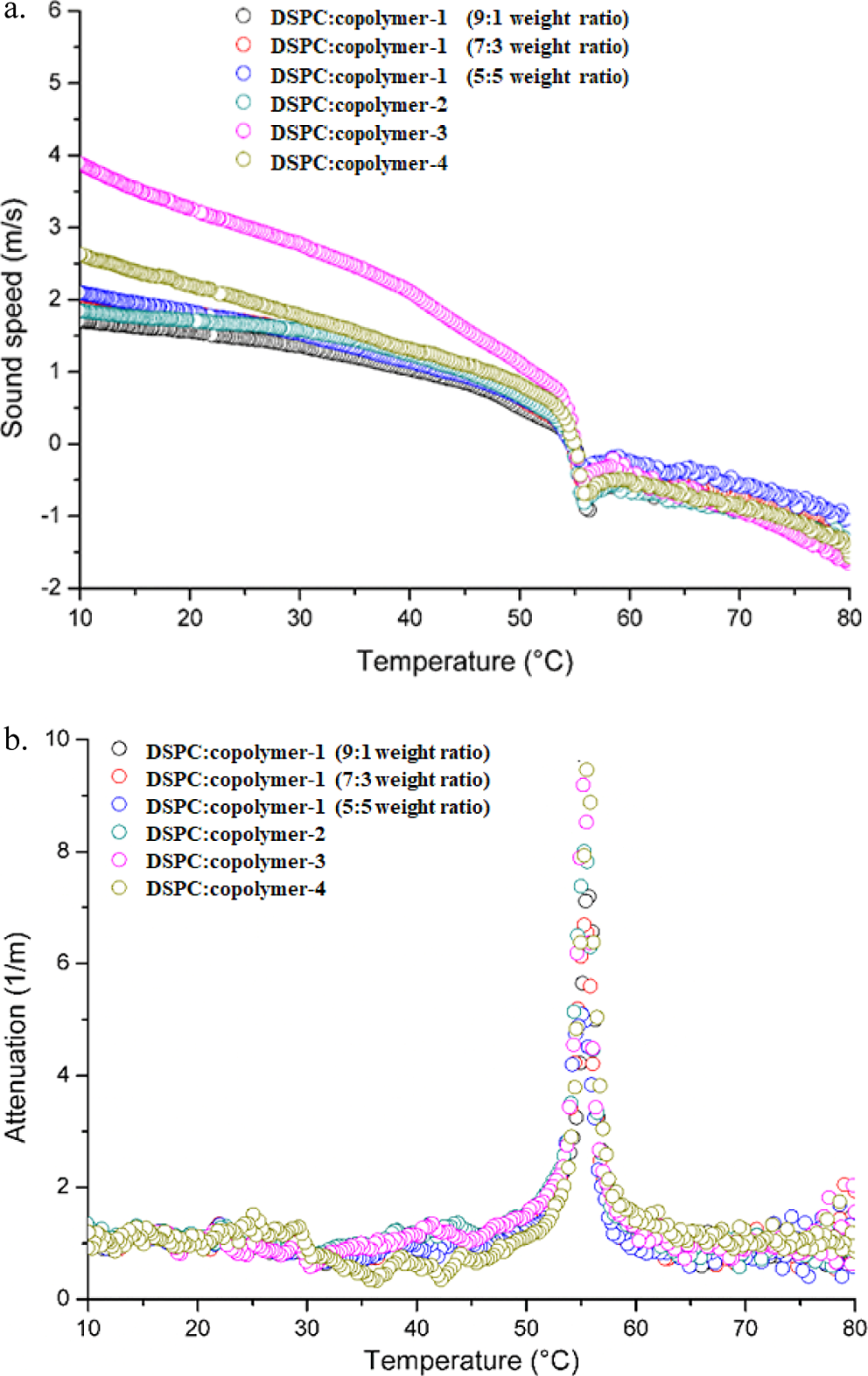
Results derived from HR-US: Sound speed and b. Attenuation vs temperature for different DSPC/P(OEGMA-*co*-LMA) hybrid systems.

The prepared hybrid structures were also probed via mDSC and HR-US in order to decipher their thermotropic behavior. MicroDSC is a well-established and highly sensitive method to characterize and even compare liposomal or hybrid colloidal dispersions—a valuable tool for generic nanomedicine/nanosimilars’ comparison.^72,73^ HR-US is also a powerful technique to study the thermal properties of biomaterials via monitoring the variation of ultrasound parameters over temperature. Namely, during the temperature-induced transition from gel to liquid state of lipids, there is also an alteration in the velocity and intensity of the propagation of the ultrasound waves through the sample.^30,35,74^ Although both techniques have extensively been used for the biophysical characterization of lipid and hybrid vesicles, to the best of our knowledge, they have never been applied for the investigation of the calorimetric features of hybrid colloidal dispersions composed of random copolymers and lipids. Their biophysical characteristics are summarized in Table 3, while mDSC traces related to heating scans as well as HR-US plots are illustrated in Figures 6 and 7, respectively.

**Table 3:** Thermodynamic Characteristics of Hybrid Lipid/Copolymer Systems, as Calculated from Microcalorimetry and High-Resolution Ultrasound Spectroscopy

sample	L/P weight ratio	mDSC		HR-US	
				(sound speed)	(attenuation)
		temperature (°C)	enthalpy (J/g)	transition temperature (°C)	
DSPC:copolymer 1	9:1	54.07 ± 0.03	0.228 ± 0.007	55.71 ± 0.27	55.42 ± 0.22
DSPC:copolymer 1	7:3	53.97 ± 0.04	0.183 ± 0.005	55.85 ± 0.21	55.58 ± 0.22
DSPC:copolymer 1	5:5	54.05 ± 0.02	0.172 ± 0.009	55.65 ± 0.31	55.32 ± 0.19
DSPC:copolymer 2	9:1	54.21 ± 0.15	0.159 ± 0.010	55.85 ± 0.32	55.28 ± 0.20
DSPC:copolymer 3	9:1	54.01 ± 0.06	0.229 ± 0.006	55.79 ± 0.09	55.20 ± 0.13
DSPC:copolymer 4	9:1	54.08 ± 0.04	0.203 ± 0.015	55.94 ± 0.23	55.52 ± 0.18

Considering mDSC measurements, we can observe that there is a main transition peak that is principally sharp and is characterized by a temperature at which the main event takes place approximately at 54 °C. This temperature is similar for all of the examined systems. The main thermal event corresponds to the transition from gel to liquid crystalline state of the lipids, and the temperature of it is in accordance with the literature regarding neat DSPC liposomes.^31^ Thus, the copolymers do not seem to have a great impact on the internal configuration of the DSPC hydrocarbon chains during the melting process. However, the area related to the transition (J/g of solution), which corresponds to the enthalpy, varies for each system, indicating different interactions and cooperativity between the biomaterials, including the copolymer chain integration into the bilayer.^29^ As a result, we can assume that the lipid-copolymer systems form morphologies with a hybrid bilayer. This is confirmed by membrane thickness measurements of cryo-TEM as well (Table 2). Namely, the incorporation of P(OEGMA-*co*-LMA)-1 at different % weights into DSPC hybrid systems demonstrates specific patterns. Even though the temperature of the main event is more or less constant and stated at 54.07, 53.97, and 54.05 °C for 9:1, 7:3, and 5:5 hybrid systems, respectively, the enthalpy is decreasing with values of 0.228, 0.183, and 0.172 J/g for the respective systems. This thermic trend is presumably owed to the van der Waals interactions between the lipid chains that are reduced and/or to the decrease of the pure lipid domain volume fraction within the hybrid membrane because of the presence of the copolymer.^52^ Considering the chemical composition of this copolymer and the fluorescence results, the OEGMA hydrophilic chains are probably in the exterior region of the structure, while the LMA segments (mainly the C12 side chains) interfere with the hydrophobic membrane, maintaining its good cooperativity. However, at a 50% copolymer weight content, the peak is a little broader, demonstrating the presence of the copolymer in the bilayer as an impurity, disorganizing the membrane during the lipid melting process.^75^ Additionally, the broadening of the peak as well as the decrease of the *T*_m_ may be the outcome of the rod-like structures that we observed in cryo-TEM images,^64^ i.e., they are morphology driven. The presence of P(OEGMA-*co*-LMA)-2 into the hybrid systems led to a more compact structure with a higher *T*_m_ in comparison to P(OEGMA-*co*-LMA)-1, probably due to more LMA segments that interact mainly hydrophobically with DSPC. However, the enthalpy value is much lower (0.159 J/g), and this could be attributed to the “reduced effective volume”, as discussed above.^76^ On the other hand, the integration of copolymers 1 and 3, which have a similar %LMA content, at the same lipid-to-polymer ratio corresponds to similar enthalpy values. This phenomenon further supports our assumption about the role of LMA segments (side chains) in the bilayer. As far as the characteristics of the lipid/copolymer dispersions comprised of P(OEGMA_500_-*co*-LMA) at different % LMA content is concerned, we can observe that the main transition temperature is similar, approximately 54 °C. DSPC:P(OEGMA-*co*-LMA)-3 has 0.229 J/g enthalpy, and DSPC:P(OEGMA-*co*-LMA)-4, which has a greater amount of hydrophobic comonomer, has 0.203 J/mol. The thermal phenomenon that is observed in the case of DSPC:P(OEGMA-*co*-LMA)-3 is illustrated by a very broad and nonsymmetric peak, which is probably reflecting a variety of mesophases.^32^ Immiscibility phenomena and domain formation are a possible explanation considering the high enthalpy value.^30^

By evaluating the aforementioned thermodynamic data in contrast to our previous DSC results for equivalent systems, we can highlight specific points. First, their state during the experimental process is different; former studies were conducted in hydrated solid systems, while the current results originate from colloidal dispersions. Therefore, the comparison refers to hybrid bilayers and hybrid particles. Moreover, the instrumentation used is different, as in the former cases, a “traditional” DSC was used and not a highly sensitive microcalorimeter. The low cooperativity that we observed for the DSPC:P(OEGMA-*co*-LMA)-3 9:1 system was not observed in the previous results. However, in both cases, we noticed similar calorimetric patterns, namely, the constant *T*_m_ and the lipid-to-polymer ratio-dependent reduction of the enthalpy. More importantly, these results strengthen our hypothesis about the location of the copolymers in the bilayer, at least regarding the lamellar structures.

As far as HR-US is concerned, this technique is able to effectively detect the main transition temperature for hybrid systems. By measuring their acoustic features, information could be rendered about the interactions of the whole material in its dispersion state with the solvent (the aqueous medium) without further dilution. The results are comparable to those of mDSC, as can be seen in Table 3, confirming the thermodynamic behavior of the hybrid colloidal dispersions. The compliance of the HR-US results to mDSC data could be accredited to the same state of the materials and the low colloidal concentration of the hybrid structures (*C* = 5 mg/mL). Figure 7a,b illustrates the change in ultrasonic parameters during temperature increases. Particularly, sound speed is reduced, while attenuation is near the baseline as it is mainly independent of temperature. However, near the temperature of the main transition, both values deviate from their trajectory, and after the completion of the thermal event, they return to their initial conditions.^31^ The results discussed above also point out the importance of using advanced, highly sensitive, and complementary characterization techniques for studying such complex soft matter hybrid systems.

## 4 Conclusions

The present study provides information about the complexity of lipid/copolymer interactions, investigating the microfluidity and the physicochemical, morphological, and biophysical properties of hybrid lipid/copolymer colloidal dispersions. Hybrid platforms of DSPC and random copolymers of the type P(OEGMA-*co*-LMA) were successfully prepared and examined via DLS, FS, cryo-TEM, mDSC, and HR-US. The different techniques confirmed the hybrid nature of the structures and the location of the copolymers in them. The random topology of copolymers and their association with phospholipids are of great importance because there are no respective hybrid lipid/copolymer systems in the literature. To the best of our knowledge, this is the second report in the literature from our research group that utilizes statistical copolymers as well as this combination of biomaterials to form hybrid structures. We believe that these lipid/random copolymer systems could be prototypical to investigate the interactions between different nature and chemistry materials for multiple applications. Gaining a deep understanding of these structures and their self-assembly could be useful in cellular membrane models due to random copolymers playing a key role in transmembrane protein mimetics. Besides, the determination of the design parameters influencing their features is useful in optimizing these promising platforms for drug delivery and personalized medicine. Depending on their size, they could be used in different routes of administration (i.e., IV, IM, nasal, per os, etc.) according to intended needs. In this perspective, the main design parameters affecting their behavior are also discussed. In particular, the random architecture of the copolymers and the hydrophobic to hydrophilic balance of the hybrid systems are highlighted as the critical factors influencing the morphology and the membrane mechanics of hybrid nanoparticles. Another interesting outcome of this work involves the correlation between the polygonal shape and the hydrophilic/hydrophobic balance, while the lipid-to-polymer ratio also has an impact on the behavior and structure of the systems.

The challenges in scaling up such complex systems and the batch-to-batch variability are well established in the literature for nanoparticle formation. The present and previous studies highlight further the need for advanced, complementary, and sensitive instrumentation and characterization techniques. To overcome these limitations, in-depth knowledge of the structure and properties of hybrid systems is needed. It is demonstrated that the crucial design parameters for the lipid/copolymer particle performance include the random architecture, the hydrophobic/hydrophilic balance, and the lipid-to-polymer weight ratio, which significantly influenced morphology, microfluidity, and biophysics. Of course, structure and properties are highly dependent on the nature of the biomaterials utilized, leading to specific interactions and thus to specific coassembly behavior, which fundamentally influences the overall behavior of the hybrid systems. One could correlate former and current results, but attention should be paid to phenomena that are multifactorial and influenced also by the size of hybrid nanostructures, such as in vivo and in vitro stability or cytotoxicity. This work gives additional insights into the behavior of lipid/copolymer hybrid nanostructures from a morphological and thermodynamic perspective and highlights the main influential design factors while punctuating the magnitude of investigating the overall performance of nanoparticles via different techniques for the desired fast clinical translation.

## Supporting Information Available

Additional information about the physicochemical and morphological results (PDF)
