# Supplementary material for: Deciphering the Lipid-Random Copolymer Interactions and Encoding Their Properties to Design a Hybrid System
Source: Langmuir. 2024 May 27;40(23):11936–46. doi: 10.1021/acs.langmuir.4c00278 (PMC11190979; doi:10.1021/acs.langmuir.4c00278)
Supplement: Supplementary file 1 — la4c00278_si_001.pdf [file la4c00278_si_001.pdf]

Supporting Information

Deciphering the lipid-random copolymer interactions and encoding their properties to design a hybrid system

Efstathia Triantafyllopoulou[a], Aleksander Forys[b], Diego Romano Perinelli[c], Anastasia Balafouti[d], Maria Karagianni[d], Barbara Trzebicka[b], Giulia Bonacucina[c], Georgia Valsami[a], Natassa Pippa[a], and Stergios Pispas[d*]*

[a] Section of Pharmaceutical Technology, Department of Pharmacy, School of Health Sciences, National and Kapodistrian University of Athens, Panepistimioupolis Zografou, 15771 Athens, Greece

[b] Centre of Polymer and Carbon Materials, Polish Academy of Sciences, Zabrze, Poland

[c] School of Pharmacy, Via Gentile III da Varano, University of Camerino, 62032

Camerino, Italy

[d] Theoretical and Physical Chemistry Institute, National Hellenic Research

Foundation, 48 Vassileos Constantinou Avenue, 11635 Athens, Greece

** Corresponding author: pispas@eie.gr (S. Pispas), natpippa@pharm.uoa.gr (N. Pippa).*

Table of Contents

S1 TABLES AND FIGURES..3

S1 TABLES AND FIGURES

Table S1. Physicochemical results of DSPC:P(OEGMA-co-LMA) hybrid systems in water for injection dispersion medium the day of their preparation.

Sample	Weight Ratio	*I (kcps)*	R_h* (nm)	PDI	$GP_{25°C}$
DSPC: copolymer 1	9:1	12290	349	0.49	0.640
DSPC: copolymer 1	7:3	7920	169	0.47	0.633
DSPC: copolymer 1	5:5	3740	294	0.48	0.620
DSPC: copolymer 2	9:1	14323	309	0.37	0.633
DSPC: copolymer 3	9:1	19581	371	0.35	0.640
DSPC: copolymer 4	9:1	14742	a) 186 (5%),<br>b) 1952 (94%)	0.33	0.653

*Precent in the parenthesis represents the weight of peak, by intensity, for systems exhibiting more than one peak.

Table S2. Physicochemical results of DSPC:P(OEGMA-co-LMA) hybrid structures in FBS:PBS (9:1 weight ratio) biorelevant dispersion medium at 37□C.

Sample	Weight Ratio	*I (kcps)*	R_h* (nm)	PDI	Number of peaks
DSPC: copolymer 1	9:1	16258	646	0.50	2
DSPC: copolymer 1	7:3	8350	370	0.49	1
DSPC: copolymer 1	5:5	4600	326	0.52	2

DSPC: copolymer 2	9:1	15613	982	0.45	2
DSPC: copolymer 3	9:1	18226	1280	0.52	2
DSPC: copolymer 4	9:1	18000	1122	0.57	1

*For systems with more than one peak R_h value corresponds to the main peak (with the highest weight of peak).

i.

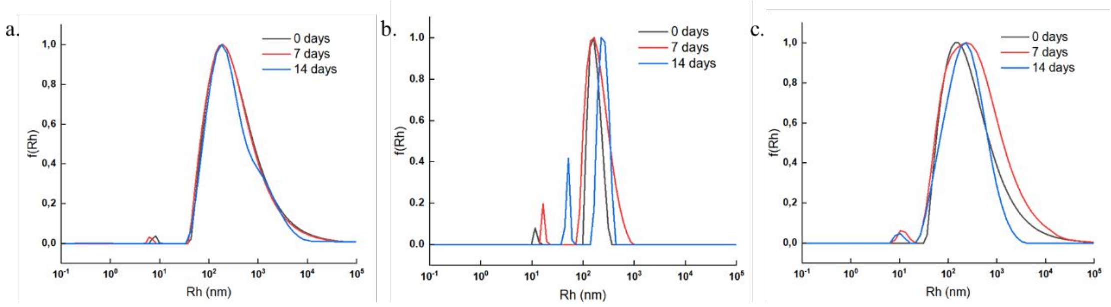

ii.

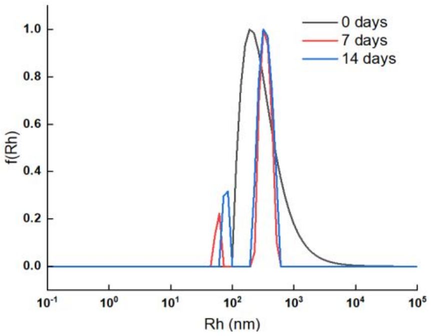

Figure S1. Size distributions from DLS of the stable for a 14-day period hybrid nanostructures: i. P(OEGMA-co-LMA)-1 at three different lipid to polymer weight ratios: a. 9:1, b. 7:3, c. 5:5, and ii. P(OEGMA-co-LMA)-2.

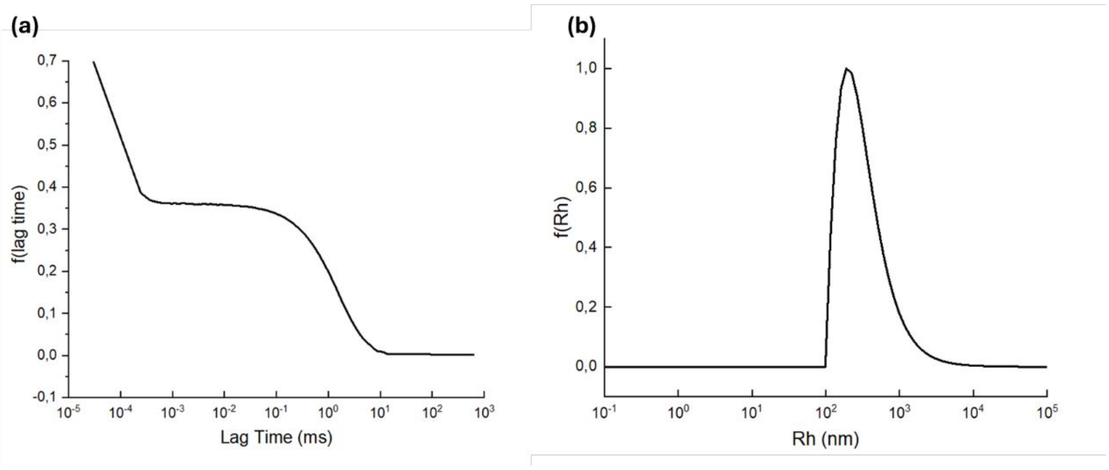

Figure S2. (a) Normalized intensity correlation function and (b) Size distribution from DLS of DSPC:P(OEGMA-co-LMA)-2 the day of its preparation as a prototype of the DLS measurements.

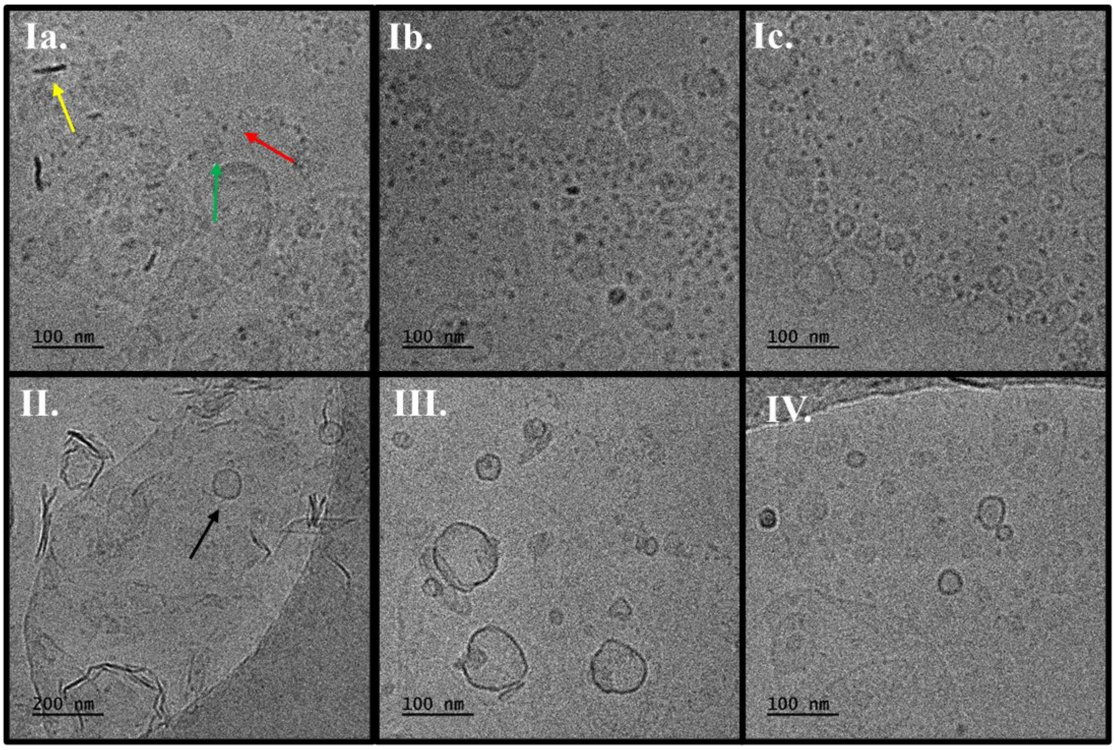

Figure S3. Additional cryo-TEM images of DSPC:P(OEGMA-co-LMA) hybrid systems of different comonomers ratio (varying % PLMA) and/or different oligoethylene glycol side chain length ($OEGMA_{950}$ or $OEGMA_{500}$): I. DSPC:copolymer-1 in different lipid to polymer weight ratios: a. 9:1, b. 7:3, and c. 5:5 ratio, II. DSPC:copolymer-2, III. DSPC:copolymer-3, and IV. DSPC:copolymer-4. The colored arrows point out a different morphology; namely small spherical particles (red

arrow), spherical or irregular shape particles with distinct membrane (green arrow), rods (yellow arrow), spherical or irregular shape vesicles (black arrow).
